# Renal effects of *Anchomanes difformis *crude extract in wistar rats

**Published:** 2015

**Authors:** Jacob Ataman E, MacDonald Idu

**Affiliations:** 1 *Department of Anatomy, University of Benin, PMB 1154, Benin City, Nigeria*; 2 *Department of Plant Biology and Biotechnology, University of Benin, Benin City, Nigeria*

**Keywords:** *Anchomanesdifformis*, *Crudeextract*, *KidneyFRAP*, *Wistarrats*

## Abstract

**Objective:** *Anchomanes difformis* is a member of the plant family Araceae which is used as a diuretic but also has other medicinal applications. This study investigates the dietary effects of *A. difformis* on the kidneys of adult wistar rats.

**Materials and Methods:** Sixteen rats were used and were weighed, before and after the experiment. All rats were randomly divided into four groups. All groups were treated with the following regimen for two weeks. The control group (A) was fed on feed mash and water *ad libitum* throughout the period. The treatment groups B, C, and D received feed mash mixed with crude extract of *A. difformis *in the following proportions: 25:75(g), 50:50(g), and 75:25(g), respectively. The kidneys of the experimental animals were histologically examined for morphologic changes.

**Results**: Results showed a significant difference (p<0.05) in the kidney weight of the treatment groups compared with the control. Histological examination of the renal tissues also showed considerable lesions such as inflammation, focal cortical and interstitial hemorrhage, and fibrosis in the treated rats compared with the control.

**Conclusion:** The current study results suggest renal toxicity with excessive consumption of *A.difformis*.

## Introduction

**Figure 1:**
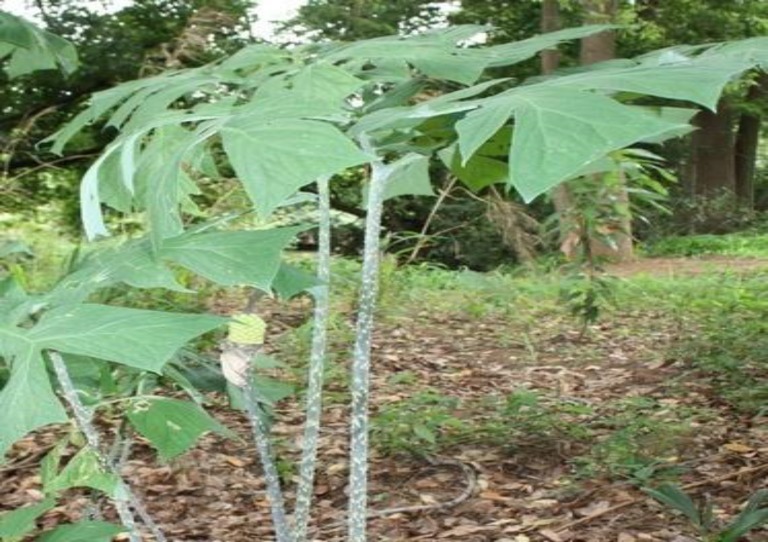
*Anchomanes difformis* (Blume) Engl. Pallidus.

*Anchomanes difformis *(Blume) Engl. Pallidus, commonly known as forest anchomanes is a plant of the family Araceae. *A. difformis *is a native plant of the African continent and grows widely in wetlands and terrestrial areas of West Tropical Africa including Nigeria, Ghana, Ivory Coast, Sierra Leone, Senegal, and Togo. *A. difformis* is a large herbaceous perennial plant with stout prickly stem (leaf petioles) of about 0.8 to 2 m high. The plant is erect on an enormous horizontal tuber, often reaching 50 to 80 cm long and 10 to 20 cm in diameter (Figure 1). It usually contains milky or watery latex, which is rarely colored. In Nigeria, *A. difformis* is locally called Olikhoror by the Bini tribe of Edo State. Despite of the toxic effects of Araceae, species of several genera are also cultivated as food plants, mainly as subsistence crops in the tropical areas. The major edible Araceae are *Colocasia esculenta* and several species of *Xanthosoma*, grown primarily for their corms and sometimes for their leaves. Most of the North American species of Araceae were historically used by the native Americans, as both food and medicine (Plowman, 1969 ▶). Further, claims from earlier report is that its leaves, stem, and roots (rhizomes) serve as food and are believed to sub-serve medicinal properties (Dalziel, 1937 ▶).

Different cultures have used these plants for different purposes. However, plants have been used as a source of medicine in virtually every culture (Gilani and Rahman, 2005 ▶.). Traditionally, *A. difformis* has been used as diuretic, antidiabetic, antituberculosis, antimalarial as well as oral and anal lesions (Joanne et al., 2009 ▶).

Plant materials continue to provide a major source of natural therapeutic remedies and play an important role in health care in many developing countries (Czygan, 1993 ▶).

Nigeria similar to many other developing countries has lost a great number of medicinal plants because in the earliest period, records of medicinal plants were virtually not available due to lack of documentation for their isolation, selection, and preparation. Every fact about potent herbal plants was passed by word of mouth from generation to generation (Kochnar, 1981 ▶). The fact that some of the available antibiotics also produce side effects and have limited efficacy (Corazo et al., 1999 ▶) has triggered researchers to discover new, safer, and more effective antibiotics from natural products especially from plants (Nitta et al., 2002 ▶). Another reason why the use of medicinal plants in the treatment of infectious diseases is common is the high cost of effective antibiotics and also drug resistance, which is very common in developing countries (Okeke et al., 1999 ▶). The high cost involved in the production of patentable chemicals and drugs have also been reported as one of the reason for a renewed interest in herbal products (Hack, 2006 ▶). 

The present necessity to investigate the safety of *A. difformis *consumption in relevance to human health necessitated this research. *A. difformis,* especially its rhizome have been reported to contain carbohydrates (77%), proteins (12%), minerals (5%), fat (0.6%), and amino acids (Busson, 1963 ▶). In Nigeria, the presence of alkaloids was also discovered in *A. difformis *(Oyetayo, 2007 ▶). Further phytochemical studies by Tchiakpe et al. (1980) ▶ revealed the presence of phenolic compounds such as catechins, epicatechins, and tannins. The rhizomes of the plant appeared to be toxic for guinea pigs on oral administration at 300 mg/kg (Tchiakpe et al., 1980 ▶). This raises the question of how safe for consumption this plant is, especially in view of its wide ethno-medicinal uses. In this study, the dietary effects of the widely used crude extract of the leaves and stem of *A. difformis* on the kidneys of Wistar rats were investigated. The kidney which is the main organ of excretion was chosen for this research because of its vital role in homeostasis and also waste product elimination (Hall, 2011 ▶).

## Materials and Methods

**Collection and Preparation of Extract**

Forest Anchomanes (*Anchomanes difformis*) was harvested from a bush at Oluku in Ovia North East Local Government Area of Edo State of Nigeria, and dried under shade to prevent the degrading of its active constituents by sun rays. The dried leaves and stems were diced into small pieces and then ground with the specimen grinding machine in the Department of Pharmacognosy, Faculty of Pharmacy, University of Benin, Benin City for authentication. The powdered form of *A. difformis* was stored in a dry container. A mixture of the powdered form of *A. difformis* and feed mash was prepared as follows: 4 kg of the plant powder was macerated in water for five days. The filtrate was concentrated using a rotary evaporator to obtain 16.1% dried crude yield. The dried crude extract was mixed with feed mash in different proportions depending on the group. Crude water extract of this sample was used in order to mimic the way the herbal practitioners use it here on their patients. Aqueous extract correspond best to the use by traditional healers (Joanne et al, 2009 ▶). This is further corroborated by previous submission that largely used by the natives, i.e., locally peeled tuber of *A. difformis* soaked in water in treating cases of dysentery (Morton, 1961 ▶). We attempted proportionate mixing with feed as the mode of administration rather than direct administration via the nasogastric tube in order to mimic its use as food (Plowman, 1969 ▶; Dalziel, 1937 ▶) and to avoid possible irritation or tissue damage.

**Experimental Animals **

Sixteen adult Wistar rats of both sexes weighing between 200-270 g were used in this study. Both sexes were employed to rule out gender bias in findings and because the plant is traditionally used by both sexes as medicine. The rats were purchased from the animal house of the College of Medicine, Ambrose Ali University, Ekpoma, Edo State, Nigeria. The rats were fed with Poultry feeds (finishers mash) produced by Edo feeds and flour mill limited, Ewu, Edo State. The animals were housed and maintained in wooden cages with the top guided by thick barb wires on its top for proper ventilation at the animals holding of the Department of Anatomy, University of Benin, Benin City. They were given water and fed liberally for three weeks until they were fully acclimatized and thereafter randomly divided into four groups of four animals per group. Group A was used as control while groups B, C, and D served as the treatment groups (Table 1).

Group A, the control group, were fed with normal feeds and water *ad libitum*, without the crude extract of *A. difformis. *Group B rats were given the highest dose of crude extract of *A. difformis*. They received 50 g of feed mash and 150 g of crude extract of the stems and leaves of *A. difformis*. Group C rats were given 100 g of feed mash and 100 g of crude extract. Group D rats received the lowest dose of extract, that is, they were fed with 150 g of feed mash and 50 g of plant extract. 

Before this administration, the weights of rats were recorded and repeated after two weeks of treatment during the experiment duration before the animals were sacrificed. The housing and handling of the animals were in compliance with the international and institutional guidelines for the care of laboratory animals in biomedical research, as promulgated by Canadian Council of Animal Care (1985). Meanwhile, approval for this work was obtained from the Department of Anatomy, University of Benin, Benin-city.

**Histopathology**

On the 14th day of extract administration to the Wistar rats, they were sacrificed by cervical dislocation following prior anesthesia using chloroform. The essence of the light anesthesia was to enable the grip on the animal before performing the cervical dislocation in line with the standard procedure as specified in the guidelines for the euthanasia of animals. While in an unconscious state, blood samples were taken directly from the heart. The kidneys were extracted and fixed in an appropriate fixative (10% formol saline) for seventy two hours. They were then placed in ascending grades of ethanol from 50% to absolute for dehydration. The dehydrated tissue was passed into xylene, a clearing agent to remove ethanol from the tissue. The cleared tissue was infiltrated in molten paraffin wax in the oven at 56^0^C to 60^0^C and passed through three consecutive changes. The infiltrated tissue was placed in molten paraffin wax in an embedding mould and allowed to cool and harden into pure paraffin tissue block which was then trimmed into shape and size and attached to a wooden chuck to aid attachment to the holder of the microtome. 

A rotary microtome was used to section the tissue at five microns (5 μm.). The floating tissues were then picked with glass slides rubbed with egg white to ease adhesion. The dried tissues on the glass slides were passed through two changes of xylene for five minutes from where they were passed through descending grades of ethanol. Staining was done using haematoxylin and eosin through the following steps: It was stained in haematoxylin for two minutes, differentiated in 1% acid alcohol and counter-stained in eosin for 2 minutes. The stained tissues were dehydrated by passing through ascending grades of alcohol, 50%, 70%, 90%, and 100% (absolute) ethanol. The sectioned tissues were then cleared in xylene. The tissues on the glass slide were mounted with cover slip using Canada balsam as mountant. The already prepared tissues were observed on the binocular light microscope for histological remarks.

**Table 1:** Treatment regimen

**Groups**	**Treatment **
A (control)	Feed mash and water *ad Libitum*.
B (highest proportion)	150g of powdered extract of *Anchomanes difformis* mixed with 50g of feed mash.
C (Intermediate proportion)	100g of powered extract mixed with 100g of feed mash.
D (lowest proportion)	50 g of powdered extract mixed with 150g of feed mash.

**Statistical analysis**

All data were expressed as mean + SEM**. **Means separation was done using the Duncan’s multiple range test (Duncan, 1957 ▶). Significant differences between the mean of each group and the control were determined using the student’s t-test at p*<0.05.*

## Results

**Physical Observation**

The weights of the Wistar rats were compared before and after the experiment (Table 2). There was a significant reduction (p<0.05) in the weights of the rats in treatment group B unlike group C (p>0.05) compared with the control. The degree of reduction was related to the amount of crude extract of *Anchomanes difformis *received. There was a slight but insignificant (p>0.05) increase in the weights of the rats in the treatment group D, comparatively. The control rats increased in their weights from initial mean weight value of 230 + 11.55 g to final mean weight value of 260 + 7.07 g at the end of the experiment. The rats in treatment group B, which received the highest dose experienced weight loss from initial mean weight value of 250 + 5.00 g to final mean weight value of 165+ 11.90 g which was significantly different (p<0.05) from the control group. The rats in group C received moderate dose of the extract and had insignificant (p<0.05) weight loss from initial mean weight of 225 + 8.66 g to final mean weight of 172.5 + 7.50 g. The rats in group D, fed with the lowest dose of extract had insignificant (p>0.05) increase in weight compared with control group, from initial mean weight of 225 + 14.43 g to final mean weight of 235.12 + 26.61 g (Table 2). 

**Histopathology**

 The renal histologic report of the rats in group A (control) showed normal features. The section revealed a detailed cortical parenchyma. 

**Table 2:** Variations in the initial and final weights in different groups of experimental animals

**Groups**	**Initial Weight**	**Final Weight**
A	230 + 11.55	260 + 7.07^a^
B	250 + 5.00	165 + 11.90^b^
C	225 + 8.66	172.5 + 7.50^a^
D	225 + 14.43	235.12 + 26.61^a^

The (a) and (b) are results of the statistical test of separation of the mean, using Duncan Multiple Range Test. There was no significant difference in groups (C) and (D) when compare with control group (A).

**Figure 2:**
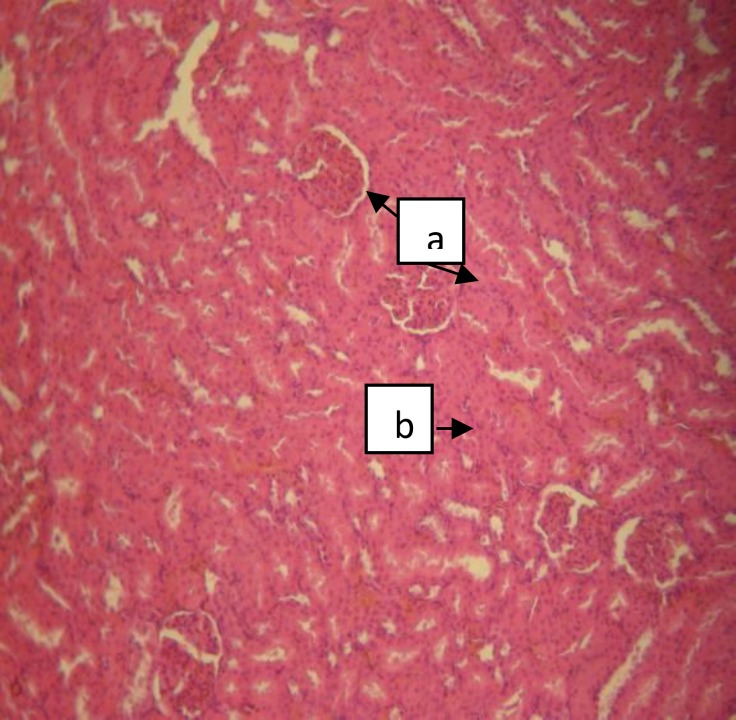
Normal kidney showing glomeruli (a) and convoluted tubules (b) [H&E x100].

Means with different alphabetic remarks were significantly different *(p<0.05).* a: compared with control group b: compared with control group with the renal corpuscles appearing as dense rounded structures and the glomeruli lying in the cups of the Bowman’s capsule (Figure 2). 

**Figure 3:**
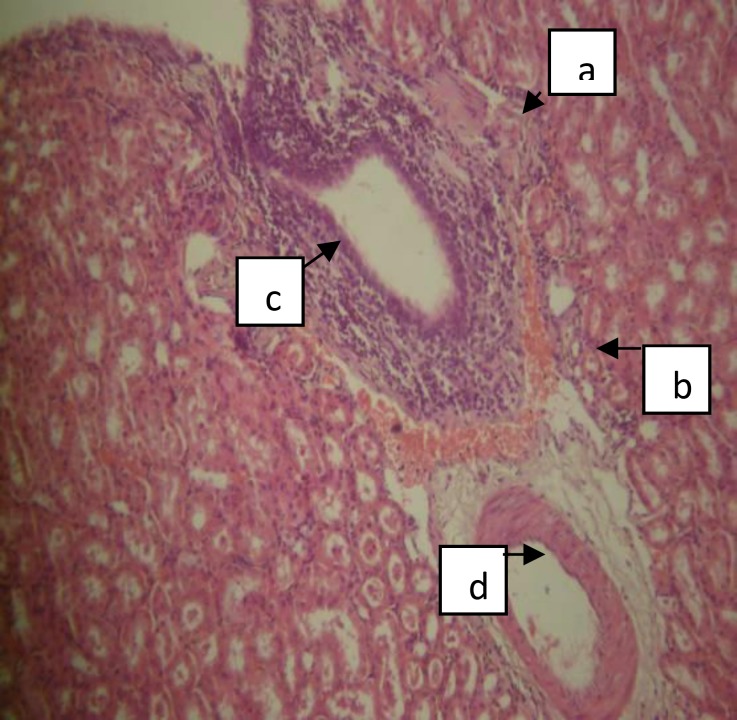
Renal histopathology of group B (Female) BF showing kidney with moderate focal interstitial fibrosis (a), hemorrhage (b), severe focal perivascular inflammation (c), and vascular hypertrophy (d) [H&E x400].

**Figure 4:**
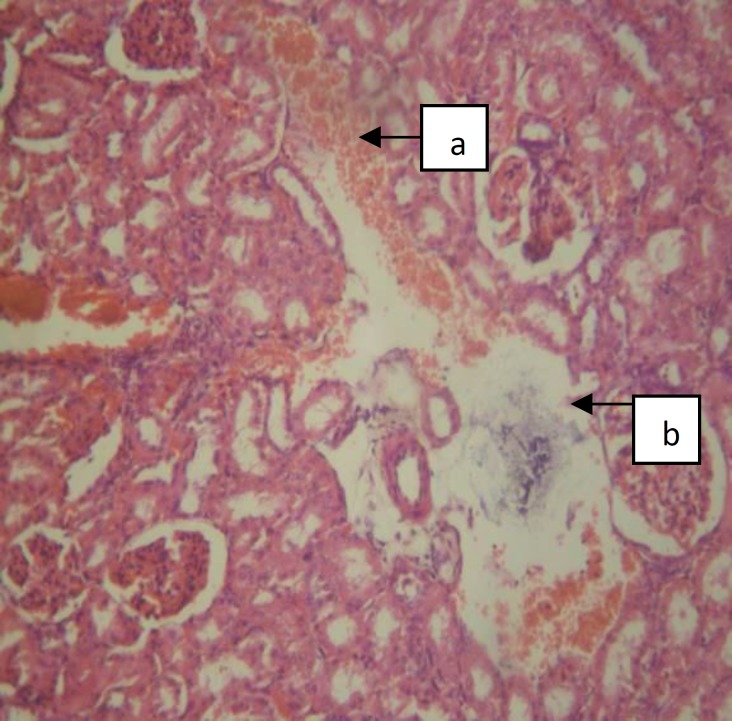
Section of group B Male (BM) kidney with severe focal interstitial hemorrhage (a) and oedema (b) [H&E x100].

**Figure 5:**
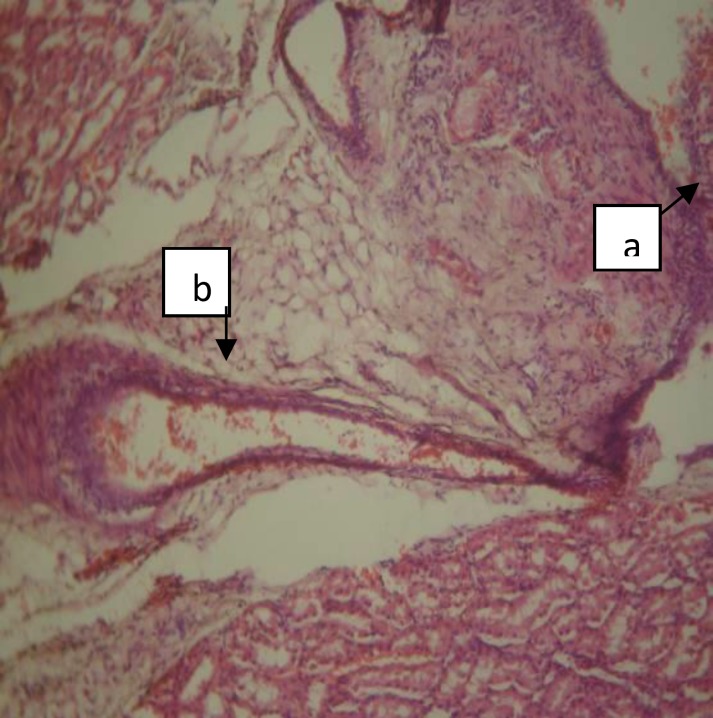
Section of group B Male (BM) kidney with severe focal interstitial fibrosis (a) and dilated and hypertrophic blood vessel (b) [H&E x100].

**Figure 6:**
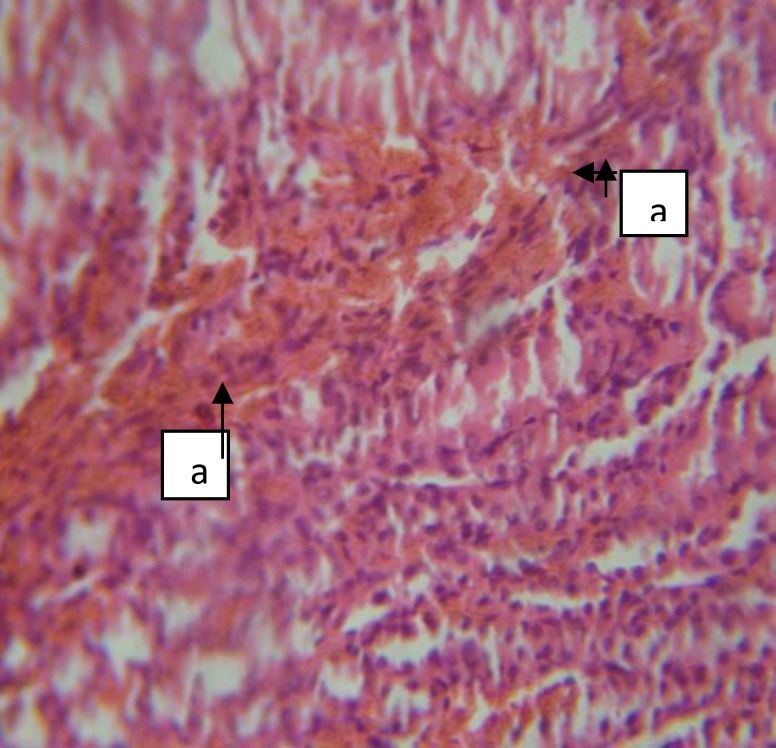
Section of group C Female (CF) kidney with severe focal cortical and interstitial hemorrhage (a) [H&E x400].

**Figure 7:**
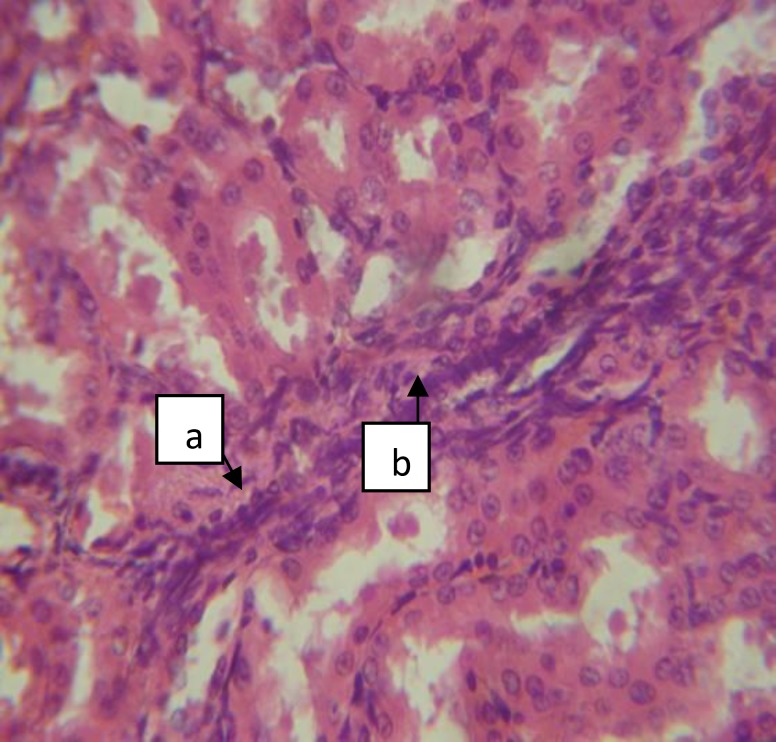
Section of group C Female (CF) kidney with severe focal interstitial fibrosis (a) and mild inflammation (b) [H&E x400].

**Figure 8:**
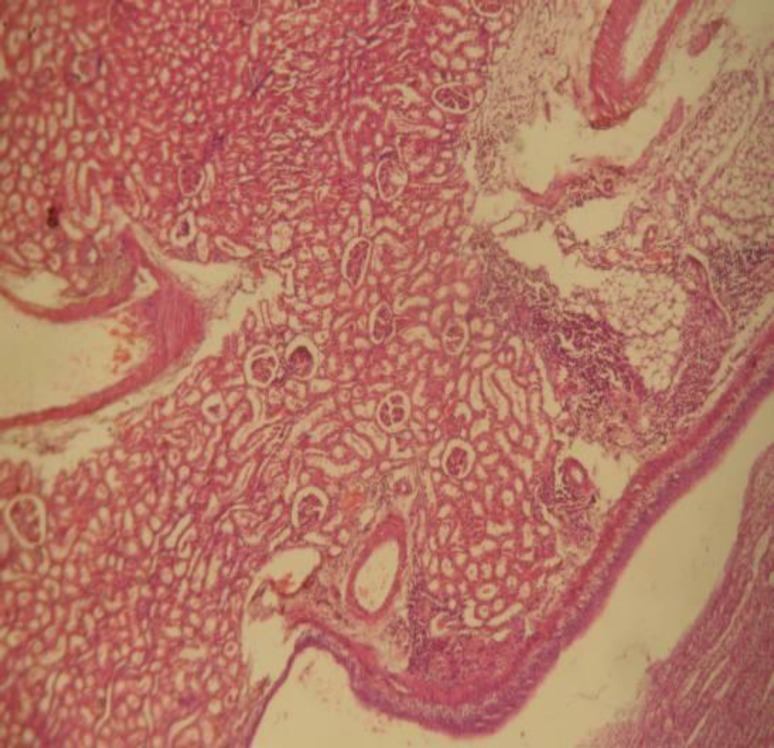
Section of group D Male (DM) kidney with focal interstitial fibrosis (a), vascular hypertrophy (b). and severe focal inflammation (c) [H&E x40].

The photomicrographs of the kidney of the rats in the treatment group B that received the highest dose of crude extract of *A. difformis *revealed areas of moderate focal interstitial fibrosis, hemorrhage, severe focal perivascular inflammation and vascular hypertrophy (Fig. 3), severe focal interstitial hemorrhage and edema (Figure 4), and also severe focal interstitial fibrosis and dilated and hypertrophic blood vessel (Figure 5). The micrographs of the kidney of the rats in treatment group C that received moderate dose of the crude extract of *A. difformis*, showed kidney with severe focal cortical and interstitial hemorrhage (Figure 6), severe focal interstitial fibrosis, and mild inflammation (Figure 7). The micrographs of the kidney of the rats in treatment group D that received the lowest dose of the crude extract of *A. difformis*, showed kidney with focal interstitial fibrosis, vascular hypertrophy, and severe focal inflammation (Figure 8)

## Discussion

This study shows that *Anchomanes difformis* which is used in the treatment of different ailments is potentially toxic to the renal tissue. Moreover, the degree of toxicity appears to be dose-dependent. The experimental design exposed the rats in group B to the highest proportion of the extract compared with the group C and D animals. However, it is not possible to entirely state that the animals in group B ingested the highest proportion of the extract. This is because as observed during study, the animals in group B and C did not consume as considerable quantity of the mixture of feed mash and extract as the animals in group D. It was observed that the group D animals ate almost as much of the admixture as group A animals ate the normal feed mash. This may have been accounted for the relative weight gain by group D animals while group B and C rats experienced weight loss. A proposed reason for this variation in the amount of feed consumption by the experimental animals is that *A. difformis *might have altered the conventional taste of the feed mash. This might have affected the appetite of the animals and caused them to consume less of the feed. Group B and C rats consumed lesser quantity of higher proportion of the extract compared with group D rats that consumed the most quantity of the extract but of lower proportion. In the end, the dose of the extract consumed by the various groups became significant enough to affect kidney damage across the groups regardless of the initial proportion of the extract in each group. The fact that group D animals experienced weight gain, though not significantly different from the control group (p>0.05) further supports the earlier suggestion of the possible effect of the extract on the appetite of the animals. Other physical signs such as agitation and hair loss were probably due to the effect of the extract on the treated animals as well. Thus, a high proportion of the extract as seen in this study may have led to varying degrees of cyto-architectural distortion in the kidney. In a situation where it has been previously reported that the rhizome is everywhere eaten in time of scarcity after special preparation (Walter and Sillans, 1961 ▶) makes this observation more imperative. That’s apart from its usefulness as food, *A. difformis* also has medicinal values is not in doubt judging from its phytochemical constituents. However, its indiscriminate use is of no beneficial purpose unless its active pharmacological components are well identified, purified, and isolated for targeted ailments. Where this is not the case, its unrestrained use might just as well be the willful introduction of harmful agents to the body. Pathological or accidental cell death is regarded as necrotic and could result from extrinsic insults to the cell such as those caused by osmotic, thermal, toxic, and traumatic effect (Farber et al., 1981 ▶). Furthermore, it is notable that kidney diseases are recently on the rise worldwide with higher incidence of renal failure (Rizwan, 2006 ▶). Worse still is the fact that in developing countries where there is so much reliance on alternative medicine, provision for renal replacement therapy is scarcely available (Lacson et al., 2005 ▶).

The results of this experiment suggest that the distortion of the cyto-architecture of the kidney by the crude extract of *A. difformis* affects kidneys of both sexes and this could have been associated with functional changes that might have been detrimental to the health of the animal. This is understandable in view of the fact that there is no morphological and functional variation between the kidneys of both sexes. In cellular distortion, the rate of progression of cyto-architectural distortion may depend on the severity of the environmental assaults. The kidney is easily susceptible to damage in view of the delicate functions it performs in maintaining homeostasis. In this study, the severity of cellular distortion appears to be dose-dependent, with chances of toxicity with even mild doses consumed over a long duration. Care should be taken when using the crude extract of *A. difformis *either as food or drug supplement. Public enlightenment discouraging unrefined herbal practices needs to be intensified, as a stitch in time could save nine.

Conclusively, we posit that administration of a high proportion of the crude extract of *A. difformis* resulted in toxic effects in the kidney. *A. difformis *therefore, though efficacious when used for therapeutic purposes has toxic potential in the kidney when used without caution, a fact that should be noted to users. 
